# Reaction inhomogeneity coupling with metal rearrangement triggers electrochemical degradation in lithium-rich layered cathode

**DOI:** 10.1038/s41467-021-25686-1

**Published:** 2021-09-10

**Authors:** Liguang Wang, Tongchao Liu, Alvin Dai, Vincent De Andrade, Yang Ren, Wenqian Xu, Sungsik Lee, Qinghua Zhang, Lin Gu, Shun Wang, Tianpin Wu, Huile Jin, Jun Lu

**Affiliations:** 1 Key Laboratory of Carbon Materials of Zhejiang Province, Institute of New Materials and Industrial Technologies, Wenzhou University, Wenzhou, Zhejiang China; 2 Department of Chemistry and Biochemistry, University of Windsor, Windsor, ON Canada; 3 Chemical Sciences and Engineering Division, Argonne National Laboratory, Lemont, IL USA; 4 X-ray Science Division, Advanced Photon Sources, Argonne National Laboratory, Lemont, IL USA; 5 Beijing National Laboratory for Condensed Matter Physics, Chinese Academy of Science, Beijing, China

**Keywords:** Batteries, Batteries

## Abstract

High-energy density lithium-rich layered oxides are among the most promising candidates for next-generation energy storage. Unfortunately, these materials suffer from severe electrochemical degradation that includes capacity loss and voltage decay during long-term cycling. Present research efforts are primarily focused on understanding voltage decay phenomena while origins for capacity degradation have been largely ignored. Here, we thoroughly investigate causes for electrochemical performance decline with an emphasis on capacity loss in the lithium-rich layered oxides, as well as reaction pathways and kinetics. Advanced synchrotron-based X-ray two-dimensional and three-dimensional imaging techniques are combined with spectroscopic and scattering techniques to spatially visualize the reactivity at multiple length-scales on lithium- and manganese-rich layered oxides. These methods provide direct evidence for inhomogeneous manganese reactivity and ionic nickel rearrangement. Coupling deactivated manganese with nickel migration provides sluggish reaction kinetics and induces serious structural instability in the material. Our findings provide new insights and further understanding of electrochemical degradation, which serve to facilitate cathode material design improvements.

## Abstract (web summary)

Electrochemical degradation is the most critical challenge for Li-rich materials. Here, the authors reveal that manganese related phase reaction inhomogeneity coupling with transition metal rearrangement triggers electrochemical degradation in lithium-rich layered cathode.

## Introduction

One of the primary challenges for modern lithium-ion batteries (LIBs) development is realizing high-energy-density cathode materials that satisfy increasing demands for long range electric vehicles. Lithium- and manganese-rich (LMR) oxides are promising candidates due to their noteworthy energy density that exceeds 900 Wh Kg^−1^ (*vs.* Li metal)^1,2^. Nevertheless, these materials suffer from serious electrochemical degradation, including voltage decay and capacity loss, which restricts real-world implementation^3,4^. Efforts to elucidate the origins of voltage decay have attributed unstable, irreversible, phase transitions from layered structures to spinel-like or rock-salt phases, induced by transition metal (TM) migration from TM layers to lithium layers, as the root cause^5,6^. Despite rising concern for capacity degradation and its close correlation to electrode reaction kinetics, its mechanisms and limitations placed on large-scale implementation remain unaddressed.

Efficient reaction kinetics are critical for electrode design and have profound effects on overall electrochemical performance^7,8^. Electrode reaction dynamics can also be influenced by many factors at multiple length scales. For example, increased cationic intermixing can block lithium diffusion pathways in the lattice structure^1,9^, and irreversible phase transformations caused by oxygen release reduce electrochemically active surfaces within cathode particles^10^. In turn, these atomic structural and chemical defects can aggravate charge heterogeneity inside the particle, inducing severe strains and stresses that exacerbate mechanical electrode deterioration^11^. Although tremendous efforts have been taken to suppress thermodynamic instability in LMR materials through methods such as cation doping^12,13^ and surface modification^14,15^, correlations between capacity degradation and the evolution of reaction kinetics remain barely studied. Given that LMR electrodes exhibit intrinsically sluggish anionic redox when compared with common ternary materials^16^, it is all the more necessary to reveal electrochemical degradation mechanisms with respect to reaction dynamics, especially at multiple length scales from atomic to particle and bulk/electrode levels.

In this work, we directly capture spatial structure evolutions in high-capacity Li_1.2_Ni_0.13_Co_0.13_Mn_0.54_O_2_ (LR-NCM) cathodes during long-term cycling using advanced synchrotron-based X-ray imaging techniques and X-ray absorption near edge structures (XANES). These techniques highlight the different behaviors of various transition metals (nickel and manganese) in the secondary particle level, revealing deactivated and correlated anisotropic reactivity for manganese and ionic rearrangement for nickel. Induced by asynchronous solid reactions, large strain and grain boundaries formed between primary crystals produce crack formations in LR-NCM particles. Subsequent structural changes at atomic levels and bulk averages were systematically studied to elucidate their contributions to electrochemical degradation, which include voltage decay, voltage hysteresis, and specially, capacity decrease. Electrochemical instability was found to be closely related to manganese deactivation and anisotropic reactions. When combined with nickel rearrangement, this instability results in electrochemical degradation of LR-NCM cathodes. These new insights into the distinct behaviors of LR-NCM transition metals elucidate the origins of capacity degradation, voltage decay, and facilitate the development of high-capacity cathode designs.

## Results

### Electrochemical properties degradation

**Fig. 1:**
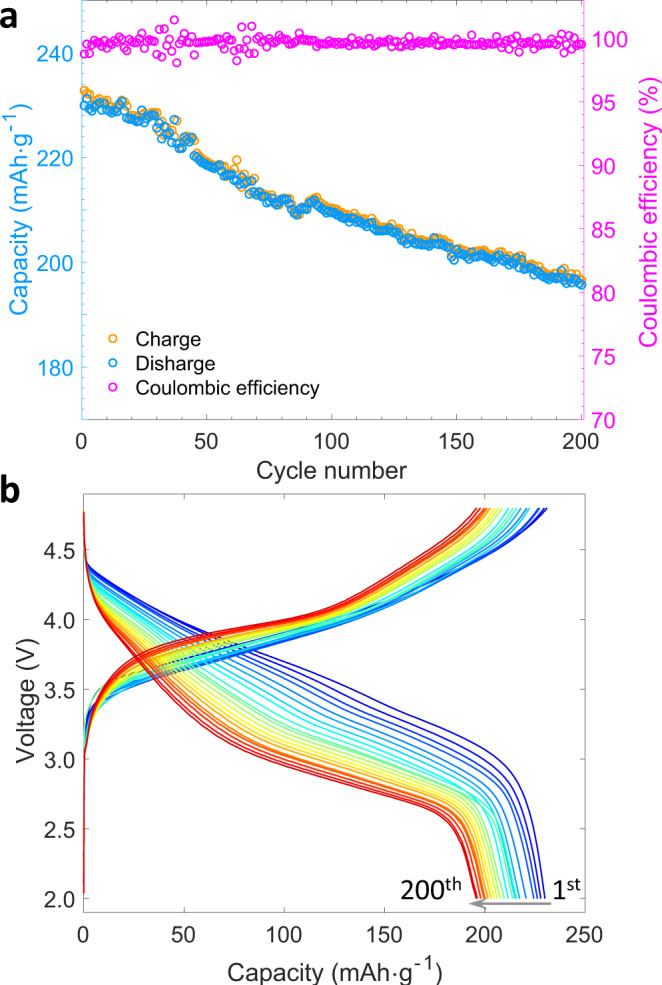
Cycling stability of LR-NCM. **a** Cycling performance of LR-NCM electrode at a current of 0.3 C. **b** Corresponding charge-discharge profiles from 1^st^ to 200^th^ cycle.

The electrochemical properties of LR-NCM material are shown in Fig. 1 and Supplementary Fig. 1. Initial charge profile (Supplementary Fig. 1a) shows a voltage plateau around 4.5 V that indicates anionic redox features in the LR-NCM material^17^. The material also exhibits dramatic capacity degradation with a discharge capacity retention of 85% as shown in Fig. 1 after 200 charge–discharge cycles at a current rate of 0.3 C (Fig. 1). As mentioned above, this capacity decrease combined with serious voltage decay limits further commercial applications. Currently, researchers primarily focus on voltage decay phenomena (illustrated in Supplementary Fig. 1b–d), which is correlated to thermodynamic changes caused by phase transformation^18^. However, key mechanisms for capacity degradation induced by structure effects and evolving reaction kinetics have been seldomly studied systematically. Herein, we employ multiple advanced characterization techniques to provide insights into the electrochemical degradation of LR-NCM.

### Revealing the reaction homogeneity in Mn and Ni

**Fig. 2:**
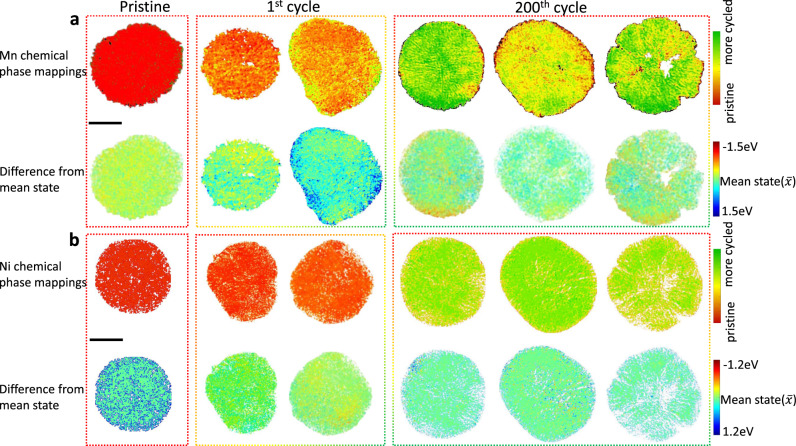
Anisotropic reaction characterized by TXM-XANES technique. 2D chemical phase mappings of LRst th NCM particles at **a** Mn K-edge and **b** Ni K-edge, respectively. Three states including pristine, 1^st^ cycle, and 200^th^ cycle, were selected. The top figure corresponds to the chemical phase mappings and the bottom row figure represents the difference from the corresponding average state. Scale bar: 5 μm.

To understand the reaction homogeneity in TMs spatially, we first employed advanced synchrotron-based full-field transmission X-ray microscopy (TXM) technique combined with XANES spectroscopy to directly observe the distribution of Ni and Mn-related chemical phases on the particle level (Fig. 2). The two-dimensional (2D) TXM-XANES mappings give the aging states mapping for each element analyzed based on the changes in XANES spectra (see detailed data processing in “Methods” and Supplementary Fig. 2). This changing in XANES spectra corresponds to the aging state of TM-based phase, which contains the electronic structural and local structural (or ligands affects around the metals) evolutions. For the Mn-related chemical phase (Fig. 2a and Supplementary Fig. 3a), chemical phase mappings (top figures) and phase difference mappings from bulk averages (bottom figures) demonstrate uniform phase distribution in the pristine state. However, phase propagation is rather anisotropic after long-term cycling and identified with distinct color coding. Prior research has proven that Mn in the pristine state is electrochemically inactive, and gradually becomes active via Mn^3+/4+^ redox during the first few cycles^19,20^. Activated Mn^3+/4+^ redox proceeds through anionic chemistry and provides excess capacity in subsequent cycles. Large red regions in particles after 200 cycles indicate reversal back to the inactive state, which we believe is one of the major reasons for electrochemical degradation. This increase in deactivated Mn ions could further aggravate charge heterogeneity and result in sluggish reaction kinetics or even isolation from the conducting network. Red regions (deactivated phase) are mostly distributed on particle surfaces and interior particles near pores, while cracks form during cycling. This phenomenon is highly correlated to chemical instability between the LR-NCM and the electrolyte as proposed by many previous works^7,21^.

As opposed to the anisotropic reactivity of Mn, Ni reactions exhibit high reversibility during long-term cycling (Fig. 2b and Supplementary Fig. 3b). Consistent color regions distributed evenly on Ni surfaces and crack formation indicate that there is no obvious chemical phase separation after 200 cycles, unlike in the 2D chemical phase mappings of Mn. This means Ni ion aging states are uniform in whole secondary particles, indicating the isotropic reactivity of Ni in LR-NCM during cycling. However, local structures around Ni ions are altered between pristine and cycled states, which could affect reactivity (discussed later in detail). The different reaction behavior between Mn and Ni recognized by TXM-XANES demonstrates that inhomogeneous reactions in Mn-related phases enhance material deterioration. Owing to the asynchronous reactivity in TMs, aggravating deactivated Mn-domains could also induce significant strain within and between crystals, leading to the formation of grain boundaries and cracks. Thus, it is essential to further investigate the chemo-mechanical changes in LR-NCM during cycling.

### Chemo-mechanical changes investigated by energy-resolved nano-tomography

**Fig. 3:**
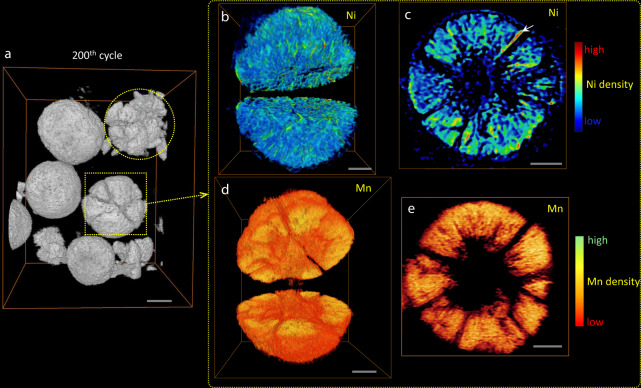
Elemental distribution visualized via 3D nano-tomography. **a** The large view of 3D nano-tomography after cycling. Scale bar: 5 μm. **b**–**e** 3D open inside view and the corresponding cross-sectional view of the selected particle in **a**. The colorbar on the right shows the concentration of the corresponding element (Ni: **b**–**c** Mn: **d**–**e**). Scale bar: 2 μm.

Three-dimensional (3D) chemo-mechanical changes were characterized by synchrotron-based energy-resolved X-ray nano-tomography (see details in the experimental section)^22,23^. Uniform Ni distribution in the whole secondary particle is confirmed by homogenous color distributions in Supplementary Fig. 4. The prepared LR-NCM exhibits spherical morphology with porous structures that are intentionally introduced to buffer volume changes during battery operation. Unfortunately, cracks still formed (Fig. 3a–e and Supplementary Movie 1) due to repeated volume changes that were coupled with large strains caused by anisotropic reactions as mentioned above. For particles after 200 cycles, the color-coded figure shows distinct color regions when compared to the pristine material, which indicates Ni segregation, rearrangement, and aggregation at red regions (Fig. 3b and Supplementary Fig. 5). These changes could induce serious irreversible phase transformation and lead to the observed voltage decay. Moreover, Ni dissolution phenomenon involving Ni^2+^ leaching into the electrolyte is also directly observed as a blue circle that appears around the spherical particle (Fig. 3c and Supplementary Fig. 5). This blue circle corresponds to low Ni concentrations observed beyond the active material, which can explain the decreased reduction peak intensity of Ni^4+/2+^ (losing capacity) in the differential capacity curves (Supplementary Fig. 1c).

In contrast, neither metal segmentation nor obvious dissolution was observed in Mn after long-term cycling (Fig. 3d, e and Supplementary Fig. 6). We selected the same particle as characterized in Ni for in-depth analysis (Fig. 3d), while another one with comparable morphology is shown in Supplementary Fig. 6. Color-coded mapping demonstrates homogenous color regions with an average concentration of Mn that indicates uniform elemental distribution inside the particles. Unlike some nickel emerging surrounding the particle as a blue circle in Fig. 3c, there is no clear manganese signals showing outside the selected particle. Consequently, we did not observe the obvious Mn dissolution phenomenon that usually shows in other Mn-based materials^24^. This suggests Mn works as a stabilizer in the solid. Compared to Ni, Mn in Li_2_MnO_3_-like domains rearranging with LiMn2 ordering remain highly stable due to average oxidation states of over 3+ even after the activation process in the first few cycles. Theoretically, Mn does not experience chemical instability because the Mn^3+/4+^ band is higher and does not overlap the O^2−^ 2*p* band^3^. This finding for high stability of Mn in the lattice can provide fundamental evidence for the capability of LR-NCM to stabilize at voltages as high as 4.8 V, which is much greater than that of traditional ternary cathode materials (NCMs).

### Atomic characterization of structural changes

**Fig. 4:**
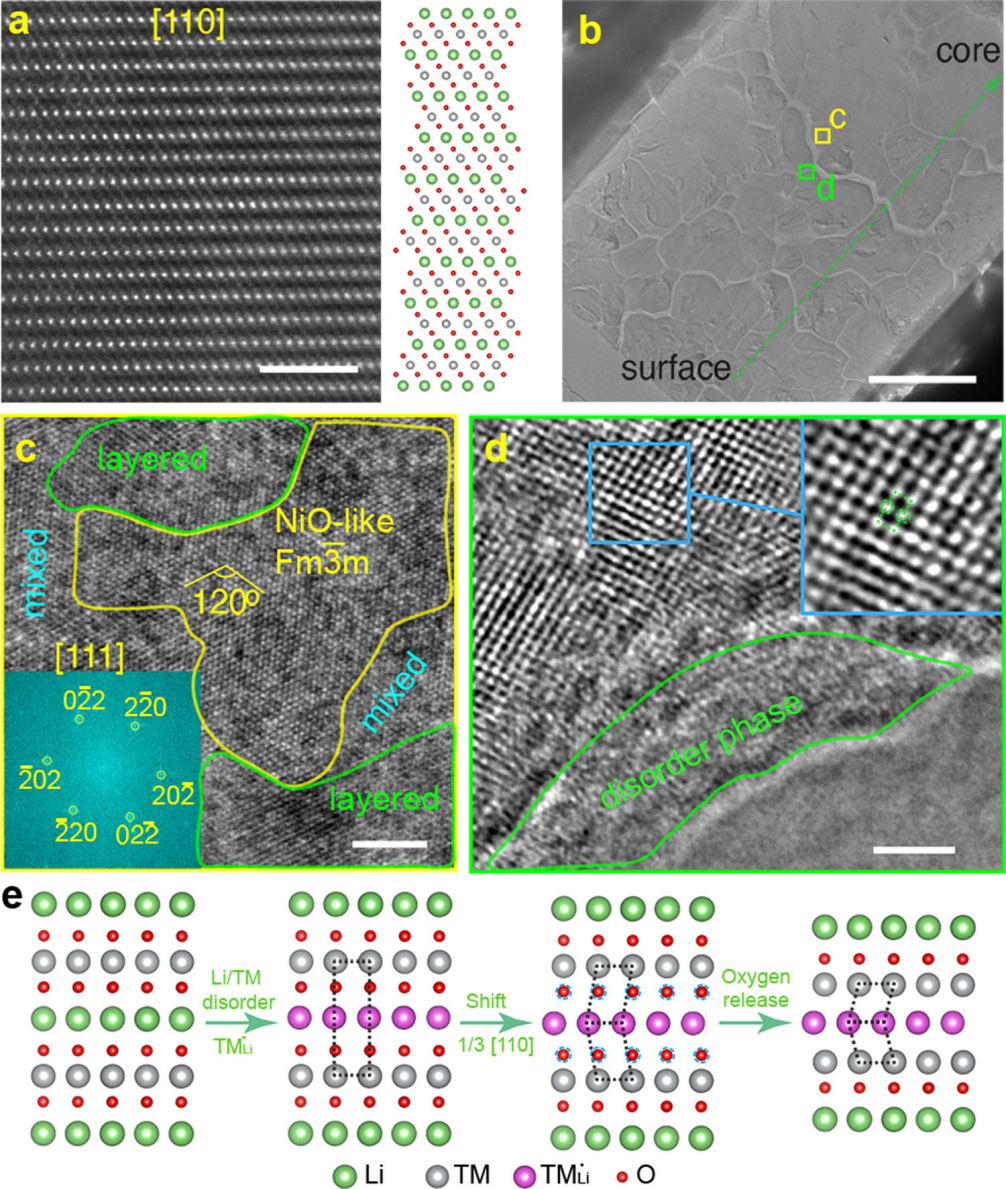
Atomic visualization of structure evolutions. **a** STEM image of pristine LR-NCM and the corresponding schematic atomic arrangement along the [110] plane. Scale bar: 2 nm. **b** Large cross-sectional view of one cycled LR-NCM secondary particle. Scale bar: 2 μm. **c**–**d** High-resolution STEM images of the selected area inside the secondary particle in **b**. Insert in **c** is FFT image of the rock-salt area. Insert image in **d** shows the magnified image of the blue region. Scale bar: 2 nm **e** Schematic illustration of structural evolutions from layered structure to rocksalt phase.

High-resolution scanning transmission electron microscopy (STEM) was performed on the particles at pristine states and after degradation to further investigate structural changes at the atomic level. Figure 4a demonstrates the perfect atomic arrangement as a layered structure for LR-NCM in the [110] plane. However, after long-term cycling, there is a consensus that serious phase transitions to spinel/rock-salt structures occur on secondary particle surfaces (Supplementary Fig. 7). Our TXM-XANES results show chemical phase deactivation even inside the secondary particle. To confirm this degradation, we further investigated the atomic structure in core particle areas (Fig. 4b). Multiple phases at different states and phase transformations from layered structure to spinel-like/rock-salt phase were observed (Fig. 4c–e). A large fully transformed rock-salt phase is demonstrated along the [111] plane with FFT imaging (Fig. 4c). A phase with severe Li/TM intermixing is also observed on other crystals in the core area (Fig. 4d). These atomic structural evolutions are closely-correlated to the stability of oxygen. When oxygen redox, O–O dimer formation along with oxygen movement can dramatically change local environment around TMs^25^, which could affect the metal rearrangement mentioned above. Agree well with previous work^26^, the irreversible phase transformation can be described in multiple gradual steps (Fig. 4e): (i) TMs migrate to vacant Li sites; (ii) the new slab shifts 1/3 along the [110] plane; (iii) spinel-like phases are formed with oxygen release. In addition, a disordered phase that is nearly amorphous appears on the particle surface, which is correlated to Ni dissolution from the solid and the formation of cathode electrolyte interfaces. Apart from these irreversible phase transformations, it cannot be neglected for the local structural evolutions in terms of high-capacity correlated superstructures in LR-NCM. Pristine material shows clear LiMn2 ordering as investigated by STEM (Supplementary Fig. 8) and the two-phase model XRD refinement (Supplementary Fig. 9 and Supplementary Table 1). Such ordering structure exists in the crystal activates the oxygen redox at high voltages, which is the primary reason for the high capacity. Therefore, the disappearing ordering structure over cycling is also considered as one of the most important factors leading to the capacity degradation^27^. This local structural ordering changes interacted with the above-mentioned phase transformations aggravate the structural irreversibility over cycling. These irreversible phase transformations accumulate into large inactive domains that hinder lithium diffusion and electron transfer, which causes degradation and electrode fatigue.

### Electronic and local structure characterized by X-ray absorption spectroscopy

**Fig. 5:**
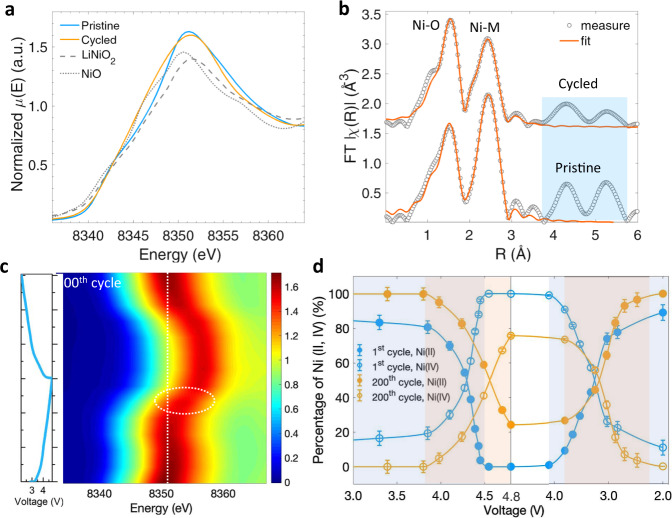
The changing reactivity of Ni. **a** Comparison of the XANES spectra and **b** the fitting results of Fourier transformed EXAFS curves of pristine and cycled electrodes at Ni *K*-edge. **c** Contour plot of *in-operando* XANES spectra at Ni *K*-edge during the 200^th^ charge-discharge processes. **d** Quantified analysis of Ni^2+4+^ redox at the first and 200^th^ cycle. Error is obtained by the linear combination fitting of each spectrum.

X-ray absorption spectroscopy at Ni and Mn *K*-edge was conducted to reveal the origin of anisotropic reactions. The XANES spectra of Ni *K*-edge remain close to that of standard NiO (Fig. 5a) except for shifts observed in the vicinity of Ni, as indicated by the white line (Fig. 5c). This indicates the valence of Ni in LR-NCM is still 2+ when cycled and further electrochemical reactions are possible. Compared to elemental Ni, Mn *K*-edge XANES spectra changes much more owing to its anisotropic reactivity over cycling as discussed above (Supplementary Fig. 10a). The absorption of pristine LR-NCM at Mn *K*-edge along the white line is similar to that of Li_2_MnO_3_, indicating valences close to 4+ which is in agreement with previously reported results^28^. However, determining the valence state of Mn ions accurately only by comparison of the profiles along the white line is difficult, owing to the complex Mn *K*-edge spectra. Part of the Mn XANES spectra of the aged electrode is close to that of MnO_2_, which demonstrates significant differences in local environments.

In order to probe local structure evolution around Ni and Mn, we further conducted quantitative analysis of the extended X-ray absorption fine structure (EXAFS) results (Supplementary Fig. 10b–d). The average bond lengths in the first two shells display no obvious variation when cycled (Supplementary Fig. 10c), except for slightly increased M–M bond lengths in the second shell. This indicates a consistent host solid structure, while small lattice extensions along the *a*(*b*) direction are explainable by increased lattice parameters determined with XRD refinement (Supplementary Fig. 9b, c). In pristine LR-NCM, the first shell of oxygen demonstrates full occupation for TMs and a coordination number (CN) for M-O of six, whereas the content in the second shell shows large differences between Ni and Mn. The second shell shows a decreased CN of Mn-M from six to three owing to the substitution of Mn by Li in the Li_2_MnO_3_-like domains. There is a consensus that oxygen release leaves many vacancies during cycling, which induces the migration of Ni ions from the center of the octahedral to the tetrahedron site. The decreased intensity of the higher order shells at distances over 3 Å represents the changed Ni ionic arrangement. In contrast, structures around Mn ions in this scattering range display more stable behavior as evidenced in the similar FT χR). The contrasting evolutions of electronic and local structure between Ni and Mn determine the degradation mechanism of Ni rearrangement and Mn deactivation, which is in good agreement with TXM-XANES and 3D nano-tomography results.

### The evolution of reaction mechanisms

We now demonstrated how electrochemical processes in LR-NCM after long-term cycling are affected by increasing anisotropic reactions and metal rearrangement. In order to further probe these effects, we investigated the electronic chemistry in Ni and Mn elements during the 200^th^ charge–discharge process by using *in-operando* XANES technique (Fig. 5c, d and Supplementary Fig. 11). Although contour plots demonstrate the full reversibility of Ni, quantitative analysis of the oxidation state as a function of voltage shows significant differences between the first and 200^th^ cycle (Fig. 5d). Ni^2+^ is fully oxidized to the highest oxidation state of 4+ at 4.5 V during the first charge, exhibiting no electrochemical activation at further high voltage. Meanwhile, the oxidation of Ni^2+^ occurs at higher average voltages in the aged electrode, which means the reaction barrier energy increased dramatically, leading to a large electrochemical reaction overpotential (*η*). In addition, average Ni oxidation states are lower than 4+ at the fully charged state and can be attributed to two factors that may also explain capacity degradation. One reason is that large *η* leads to serious voltage hysteresis and sluggish reaction kinetics that result in only partial Ni^2+^ oxidation at the same cutoff voltage of 4.8 V. Another is that a fraction of Ni^2+^ is involved in the formation of electrochemical inactive NiO-like phases. The discharge process demonstrates contrasting behavior, where the reduction of Ni^4+^ in the aged electrode takes place at lower voltages compared to the first cycle. Interestingly, although Mn is partially deactivated after cycling, the overall reaction showed active Mn^3+/4+^ redox with high reversibility (Supplementary Fig. 11). The growth in the intensity of the second pre-edge peak corresponds to the emptying of electrons in the e_g_ level, which suggests the oxidation of Mn^3+^ to Mn^4+^ ^29^. Unlike during the first cycle, the pre-edge peaks of *in-operando* Mn *K*-edge XANES spectra during the whole 200^th^ cycle change around one peak state as shown in Supplementary Fig. 10, indicating a small range valence evolution. This phenomenon confirms the presence of electrochemically deactivated Mn after degradation, which explains LR-NCM capacity loss.

**Fig. 6:**
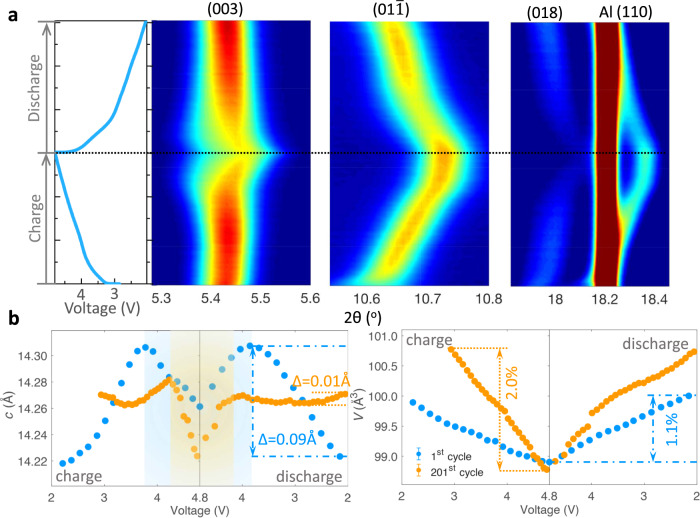
Comparison of phase evolutions. **a** Contour plot of *in-operando* XRD patterns during the 200^th^ cycle. Left figure shows the corresponding charge-discharge curves. Red to blue represents the decreasing peak intensity. **b** Comparison of the lattice parameters between the 200^th^ cycle and the first cycle after the electrode activation at a low rate of 0.1C. The lattice parameters (*c* and *V*) with errors were obtained by the corresponding *in-operando* XRD refinement.

*In-operando* long-duration synchrotron XRD was carried out on the LR-NCM cathode at the first and 201^st^ cycles to understand the evolution of the aged LR-NCM (Fig. 6a and Supplementary Figs. 13, 14). During initial charging (003) reflection shifts gradually to lower angles, which corresponds to increase in *c*-spacing (Fig. 6b) induced by the growing repulsive force between two oxygen layers after lithium extraction. However, this process is put off to even higher voltages in the aged electrode (Fig. 6b) due to the large energy barrier caused by increased cationic intermixing as proved above. Slightly splitting of (015) reflection (Supplementary Fig. 14b) also proved the restricted H1–H2 transformation, indicating the losing reactivity in TMs. Given the small changes in the diffraction patterns, further follow-up studies to clarify this peak splitting is necessary to clearly demonstrate the phase transition. *C-axis* values correspond to state-of-charge (SoC) changes, and smaller variations at low voltages during the 201^st^ cycle explain the origin of capacity loss as the result of metal migration. The narrow structural evolution at high-voltage range (yellow area in Fig. 6b) caused by the large overpotential in the aged electrode demonstrates another part of capacity degradation. In addition, the unit cell volume (*V*) exhibits nearly two times increase in magnitude for the aged electrode (2.0%) than in the first cycle (1.1%). This large volume evolution when cycled could further aggravate stress inside the solid, resulting in growing grain boundaries and crack formations as observed by 3D nano-tomography. Note that we only applied one-phase model in *in-operando* XRD patterns refinement to avoid the deviations arising from two-phase model fitting complex (Supplementary Fig. 15). The average lattice parameters were obtained by this method, which would not affect the conclusion of sluggish reaction kinetics in aged electrode. These *in-operando* XANES and XRD results suggest the electrochemical degradation of Li-rich layered oxides originate from the asynchronous reactivity between various TMs and chemo-mechanical instability.

### Reaction kinetics

**Fig. 7:**
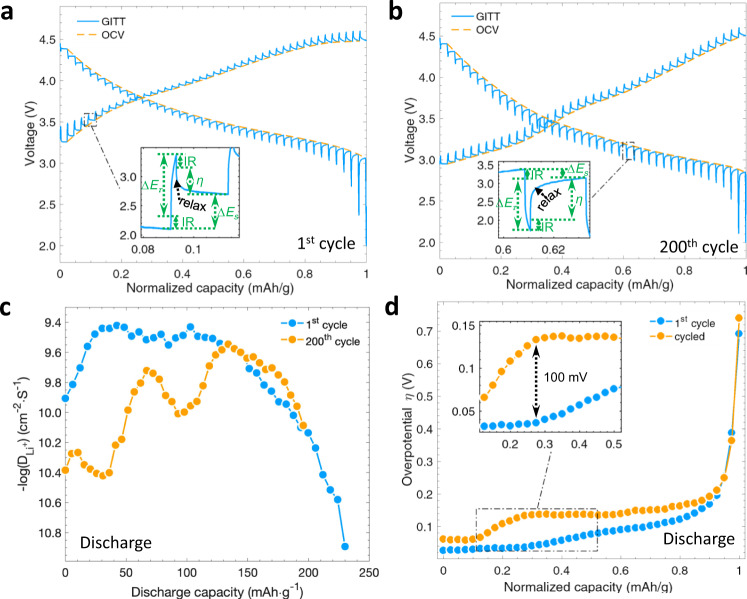
Evolutions of reaction kinetics. GITT measurements at **a** the first cycle after low-rate activation and **b** after 200 electrochemical cycles. The open circuit voltage (OCV) obtained after 120 min relaxation at each current pulse was connected with yellow dashed lines. Inserts were the close-up view of the corresponding one current pulse process. IR drops, changes in steady-state voltage (Δ*E*_*s*_) and the total battery voltage (Δ*E*_τ_) change at one single-step constant pulse were marked to show the main parts that contributed to Li^+^ diffusion coefficient. **c** The corresponding D(Li^+^) and **d** reaction overpotential (*η*) of the electrode at different states during the first and 200^th^ discharge process.

The electronic and physical structural changes investigated above can unequivocally induce sluggish reaction kinetics and lead to electrochemical degradation. Galvanostatic intermittent titration techniques (GITT) and electrochemical impedance spectroscopy (EIS) were used in the first cycle and after long-term cycling (Fig. 7 and Supplementary Figs. 16, 17) to quantify kinetic evolution. The parameters of the charge-transfer resistance, Li^+^ diffusion coefficient (${D}_{{{{{{\rm{L}}}}}}{{{{{{\rm{i}}}}}}}^{+}}$), and reaction overpotential (*η*) were quantified as a function of capacity to investigate electrochemical kinetic variations. The significantly increased semicircle in the high frequency region of EIS curves after cycling (Supplementary Fig. 16) indicates increased electrochemical reaction resistance in the LR-NCM electrode. As a function of discharge capacity, the ${D}_{{{{{{\rm{L}}}}}}{{{{{{\rm{i}}}}}}}^{+}}$ (Fig. 7c) and *η* (Fig. 7d) show distinct behaviors at different cycles over the whole Li^+^ extraction process. Upon the 200^th^ discharge, ${D}_{{{{{{\rm{L}}}}}}{{{{{{\rm{i}}}}}}}^{+}}$ values drop sharply, and the overpotential *η* increases up to 100 mV. This growing polarization and lithium diffusion decrease are highly correlated to the chemical and electronic structural changes that correspond with the evolution of reaction inhomogeneity as shown above. In this study, we, therefore, reveal the origin of electrochemical degradation and relate structural changes to it with respect to reaction kinetics.

## Discussion

**Fig. 8:**
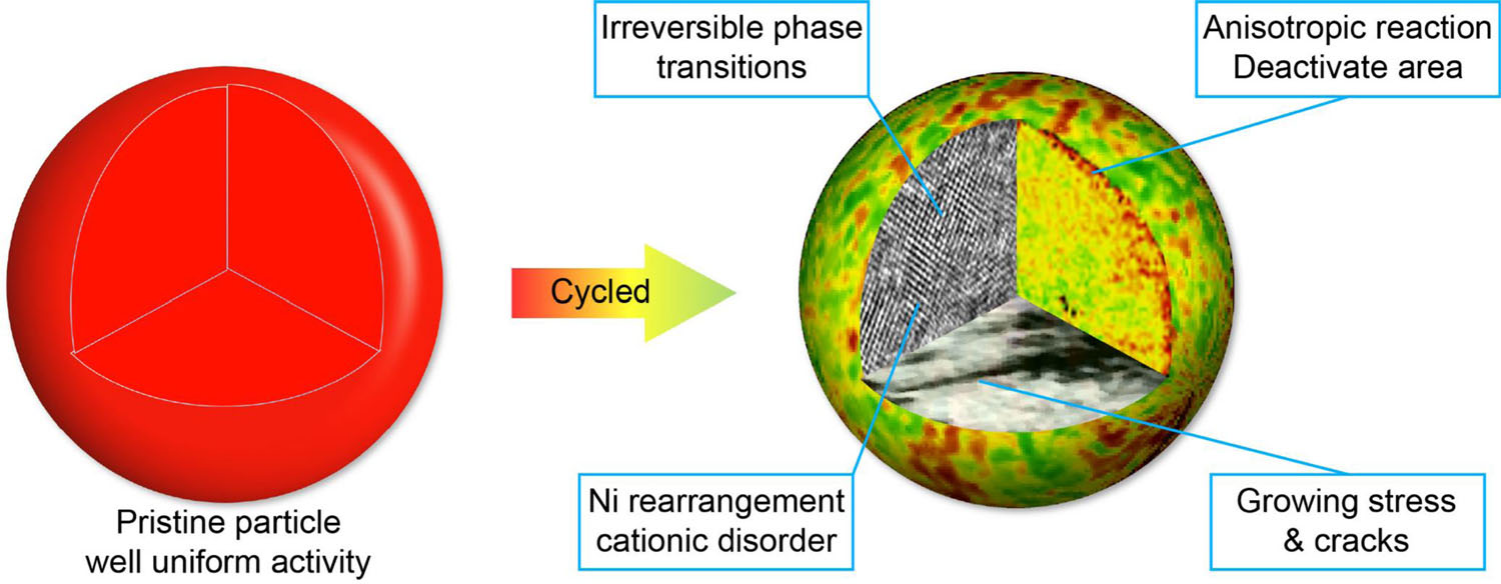
Schematic illustration of the degradation mechanism.

In summary, the spatial structure, which includes spatial electronic and chemo-mechanical structure and spatial electrochemistry, at multiple length scales ranging from atomic to 3D bulk averages play a major role in battery electrochemical degradation^22^. Current understanding of spatial reactivity in LR-NCM material reveals the deactivation of manganese and rearrangement of nickel that results in anisotropic particle reactivity (Fig. 8). This inhomogeneous reactivity leads to small localized lithium diffusion coefficients and augments reaction impedance, which is closely-correlated to electrochemical performance^30^. In turn, sluggish local reaction kinetics could further aggravate local charge inhomogeneity, resulting in increased strain inside the material and eventual mechanical deterioration of the electrode. Non-uniform lithiation states in the material arise from anisotropic reactivity in various transition metals, which causes structural and electrochemical degradation. Anisotropic reactivity and metal rearrangement were therefore revealed as the major reasons for electrochemical degradation in LR-NCM material.

Based on our findings, surface modification combined with doping could greatly resolve electrochemical degradation in LMR. Though, surface modification or doping alone may not fully suppress both capacity loss and voltage decay issues^13^. For example, electrochemical degradation was still observed in LMR materials coated with only MnO_2_^31^ or LiFePO_4_^32^_._ However, surface modification with partial doping near the surface area leads to greatly enhanced voltage performance and cycle stability due to the formation of a stable, uniform, electrochemically active surface^16,33^. A new dual particle doping with surface architecture approach is on the way in our group to address these electrochemical degradation problems in LMR material. Nevertheless, this study offers valuable insights into battery material degradation mechanisms at multiple length-scales for LMR material, which is critical for fundamental research and realization of practical applications.

## Methods

### Material preparation

The Li- and Mn-rich cathode material was prepared through a solid phase reaction between precursor and LiOH at 900 °C for 20 h in air atmosphere. Mn_0.666_Ni_0.167_Co_0.167_CO_3_ precursor was prepared with a traditional co-precipitation method, where MnSO_4_·H_2_O, NiSO_4_·H_2_O, and CoSO_4_·H_2_O material, at a molar ratio of 4:1:1, were dissolved in deionized water to obtain a 2.0 M TM solution. This solution was then slowly pumped into a 2 L tank and continuously stirred under nitrogen atmosphere. The solution pH value was maintained at 7.5 by the Na_2_CO_3_ and NH_3_·H_2_O solutions, and reacted for 12 h to obtain Mn_0.666_Ni_0.167_Co_0.167_CO_3_ precursor.

### Electrochemical measurements

The electrode was prepared with 80 wt% active material, 10% polyvinylidene fluoride, and 10% carbon black in N-methyl-2-pyrrolidinone and coated onto aluminum current collectors. 2032-type coin cells were assembled in an argon-filled glovebox (O_2_ and H_2_O level last than 0.5 ppm) using 1 M LiPF6 in a mixture of ethylene carbonate and dimethyl carbonate (1:1 by volume) as the electrolyte. All the batteries used in this work were tested by the MACCOR battery cycler at room temperature. The cycle performance of LR-NCM was measured at acurrent of 0.3 C for 200 cycles right after three activation cycles at a low rate of 0.1 C.

### Physical characterization

In-situ X-ray absorption spectroscopy experiments were conducted at beamline 9-BM-C and 12-BM-B, advanced photon source (APS), Argonne National Laboratory (ANL), at Ni and Mn *K*-edge through the transmission mode. XAS data were normalized, Fourier transformed, and analyzed using Athena and Artemis packages. In-situ X-ray diffraction experiments were performed on the LR-NCM electrode during the 200^th^ cycle at beamline 17-BM with the X-ray wavelength of 0.4526 Å. The XRD patterns were integrated and analyzed using GSAS-II packages. A coin cell with 3 mm Kapton window was used for in-situ XANES and in-situ XRD measurements (see the cell design in Supplementary Fig. 18). The in-situ experiments should be carefully design and setup to avoid the detrimental effects caused by the non-conductivity Kapton window on the in-situ cell. In this study, we used the normal electrode design with aluminum as the current collector to achieve high electric conductivity and to eliminate the detrimental effects. Advanced in-situ battery cell AMPIX that developed by APS, ANL, would also much help to remove these effects^34^. Full-field transmission X-ray microscopy data were collected at beamline 32-ID-C and the experimental details for 2D chemical mappings and 3D nano-tomography are described as in the following section.

### Full-field transmission X-ray microscopy

#### 2D TXM measurement and data analysis

The ex-situ 2D TXM-XANES chemical mappings were collected at both Ni (8333 eV) and Mn (6539 eV) *K*-edge^35^. To record the phase transformations in the material, a full series of XANES images were collected on the material at pristine and after cycled. The full XANES images were collected by scanning *K*-edge (e.g., Mn, 6539 eV) from 6519 to 6639 eV with an energy step size of 1 eV, which generated 1024 × 1024 XANES spectra, corresponding to ~30 nm output pixel size. The exposure time for each XANES image was 2 seconds. The background images collected at each energy were applied on all the corresponding XANES images. Then, we can extract the full XANES spectrum (X-ray intensity vs energy) for each pixel. Based on Beer’s Law, the attenuation coefficient *µ* for the given phase and thickness *t* could be defined as:

1 $$\frac{I}{{I}_{0}}=\exp (-\mu ({{{{{\rm{E}}}}}})t)=\,\exp (-{\mu }_{{{{{{\rm{pristine}}}}}}}{t}_{{{{{{\rm{pristine}}}}}}})\cdot \exp (-{\mu }_{{{{{{\rm{cycled}}}}}}}{t}_{{{{{{\rm{cycled}}}}}}})$$

where *I*_*0*_ is the incident X-ray intensity and *I* is corresponding X-ray intensity after the attenuating phase. Note that *µ* is a function of energy and can be attributed to the two pristine and cycled states.

The scaled *-ln(I/I*_*0*_*)* at each pixel was then fitted by the linear combination of two *µ* values. The ratio of the weighting factor is analogous to the thickness fraction and therefore represents their volume fraction.

2 $$-\,{{{{\mathrm{ln}}}}}\left(\frac{I}{{I}_{0}}\right)={\mu }_{{{{{{\rm{pristine}}}}}}}{t}_{{{{{{\rm{pristine}}}}}}}+{\mu }_{{{{{{\rm{cycled}}}}}}}{t}_{{{{{{\rm{cycled}}}}}}}$$

The spectrum fitting was carried out by minimizing the measure of misfit (*R* value) for each spectrum at each pixel, which is defined as:

3 $${{{{{\rm{R}}}}}}=\mathop{\sum }\limits_{{{{{{\rm{Ei}}}}}}}^{{{{{{\rm{Ef}}}}}}}{({{{{{\rm{dataE}}}}}}-{{{{{\rm{refE}}}}}})}^{2}/\mathop{\sum }\limits_{{{{{{\rm{Ei}}}}}}}^{{{{{{\rm{Ef}}}}}}}{{{{{{\rm{dataE}}}}}}}^{2}$$

where Ei and Ef are the beginning and ending scan energy (Ni: 8313–8413 eV, Mn: 6519–6619 eV), respectively. dataE is the normalized spectrum for the given energy E at each pixel, and refE is the possible fitting reference value that is a linear combination of X-ray attenuation of the pristine and cycled material. Detailed experimental setup and principles and data processing of 2D TXM-XANES mappings can be found in Supplementary Fig. 2.

#### 3D nano-tomography

3D elemental distribution on the secondary particles was obtained by calculating the difference between below and above X-ray absorption edge. All the 3D nano-tomography data were collected at the energies of below and above absorption edge (Mn: 6530 eV and 6565 eV, Ni: 8313 eV and 8360 eV) to resolve the Mn and Ni distribution. The 3D distribution of TMs can be achieved by subtracting the data of low energy from the data of above absorption edge. 1021 projection images were collected for each sample over an angular range of 180° from −90° to 90° enabling high spatial resolution of sub-40 nm. The 3D nano-tomography data were reconstructed from these two-dimensional projections with in house code running on Python.

## Supplementary information

Supplementary Information

Description of Additional Supplementary Files

Supplementary Movie 1

## Data Availability

All the relevant data are available within the paper and its Supplementary Information file or from the corresponding author upon reasonable request.
