# Supplementary material for: Reaction inhomogeneity coupling with metal rearrangement triggers electrochemical degradation in lithium-rich layered cathode
Source: Nat Commun. 2021 Sep 10;12:5370. doi: 10.1038/s41467-021-25686-1 (PMC8433364; doi:10.1038/s41467-021-25686-1)
Supplement: Supplementary file 1 — Supplementary Information [file 41467_2021_25686_MOESM1_ESM.pdf]

Supplementary Materials

Reaction inhomogeneity coupling with metal rearrangement triggers electrochemical degradation in lithium-rich layered cathode

Liguang Wang,[1,2] Tongchao Liu,[3] Alvin Dai,[3] Vincent De Andrade,[4] Yang Ren,[4] Wenqian Xu,[4] Sungsik Lee,[4] Qinghua Zhang,[5] Lin Gu,[5] Shun Wang,[1] Tianpin Wu,[4] Huile Jin,[1*] and Jun Lu[3*]*

1. Key Laboratory of Carbon Materials of Zhejiang Province, Institute of New Materials and Industrial Technologies, Wenzhou University, Wenzhou, Zhejiang 325035, China
2. Department of Chemistry and Biochemistry, University of Windsor, Windsor, ON N9B3P4, Canada
3. Chemical Sciences and Engineering Division, Argonne National Laboratory, 9700 South Cass Avenue, Lemont, IL 60439, USA
4. X-ray Science Division, Advanced Photon Sources, Argonne National Laboratory, 9700 S Cass Ave., Lemont, IL 60439, USA
5. Beijing National Laboratory for Condensed Matter Physics, Chinese Academy of Science, Beijing 100190, China
Correspondence and requests for materials should be addressed to: junlu@anl.gov (J. Lu); twu@anl.gov (T. Wu); and huilejin@wzu.edu.cn (H. Jin)

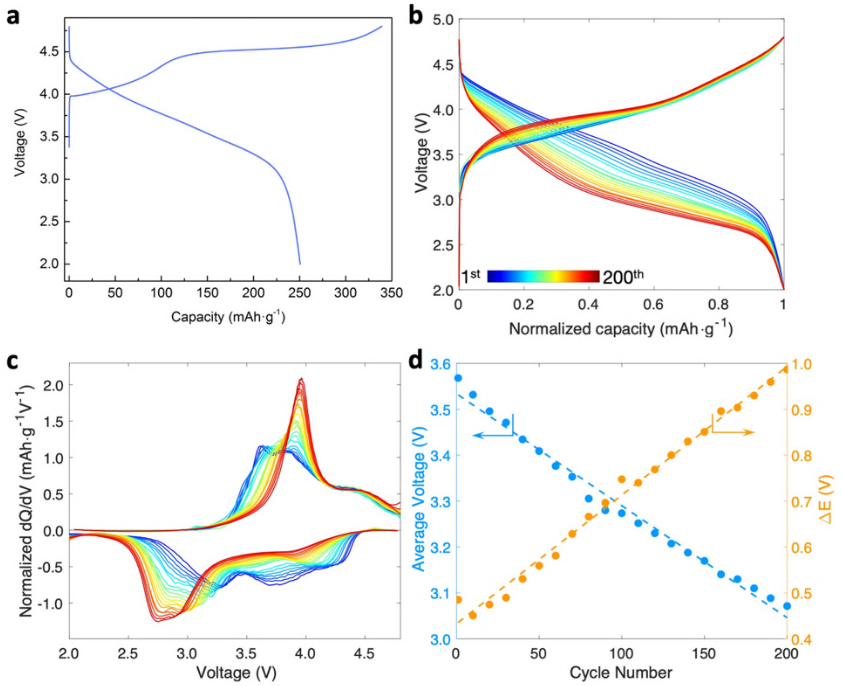

Supplementary Fig. 1. Electrochemical performance of LR-NCM. a, Initial charge-discharge profiles. **b**, Normalized charge-discharge profiles and **c**, normalized differential capacity curves from 1st to 200th cycle at a current rate of 0.3C. **d,** Average voltage changes during discharge and voltage differences (ΔE) between charge and discharge.

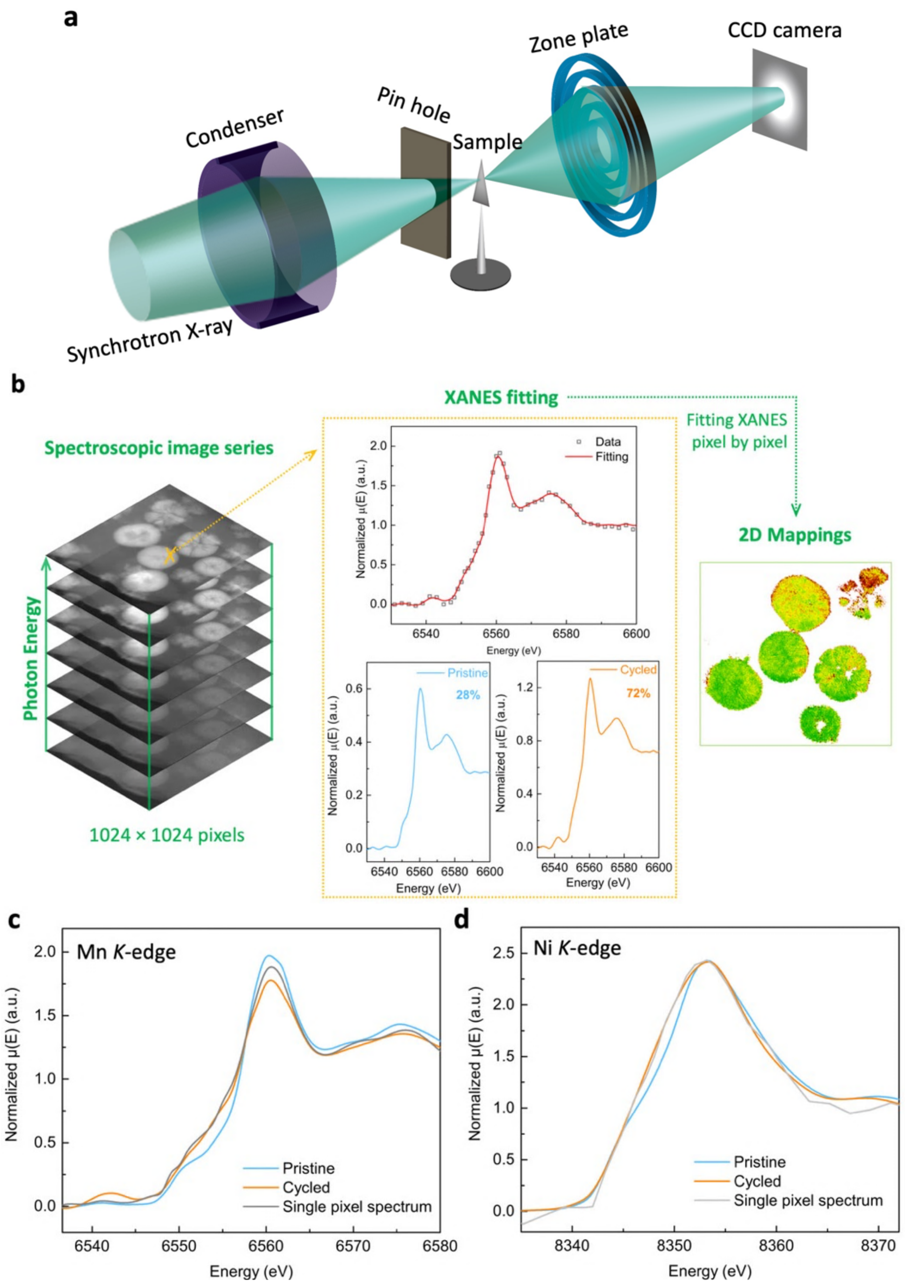

Supplementary Fig. 2. Experimental setup and data processing for 2D TXM-XANES mappings. **a**, Schematic illustration of 2D TXM measurements. **b**, Principles and data processing of 2D TXM-XANES mappings. The comparison of single pixel XANES spectrum extracted from 2D TXM spectroscopic images series at **c**, Mn K-edge and **d**, Ni K-edge.

2D TXM-XANES

Firstly, a series of full field spectroscopic images with transmission mode were collected by scanning energy across transition metal (Mn and Ni) *K*-edge with an energy step size of 1 eV. These spectroscopic images can generate 1024×1024 XANES spectra, corresponding to ~30 nm output pixel size. Then, we can extract the full XANES spectrum (X-ray intensity *vs* energy) from each pixel (Supplementary Fig. 2c-d). Based on Beer's law, The XANES spectrum can be

processed to achieve the normalized attenuation coefficient μ function. The composition of the pixel can be obtained by the linear combination fitting with the two XANES spectra at the pristine and cycled states. Fitting the XANES spectrum pixel by pixel in the whole spectroscopic image can finally get 2D TXM-XANES mappings. 2D TXM-XANES chemical phase mappings are mainly determined by the position of the half peak height.

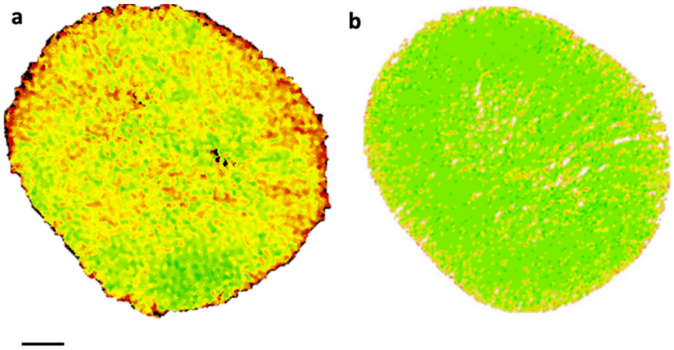

Supplementary Fig. 3. Magnification TXM-XANES mappings of the selected particle. 2D chemical phase mappings of LR-NCM particles at **a**, Mn *K*-edge and **b**, Ni *K*-edge, respectively. Scale bar: 2 μm.

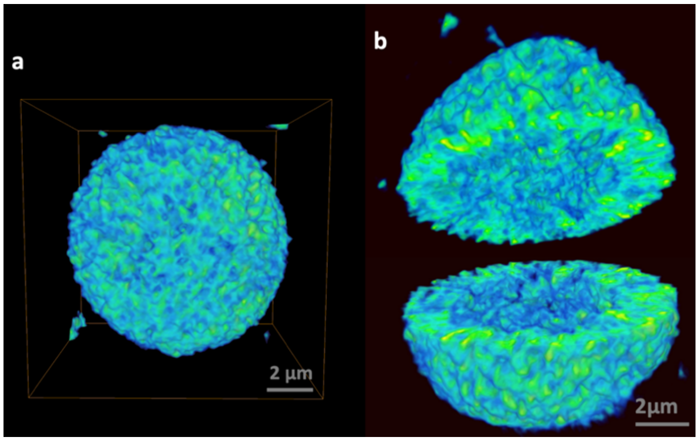

Supplementary Fig. 4. Elemental distribution observed via 3D nano-tomography. a, 3D nano-tomography and **b**, open 3D view of Ni distribution in the prepared pristine LR-NCM particle. Scale bar: 2 µm.

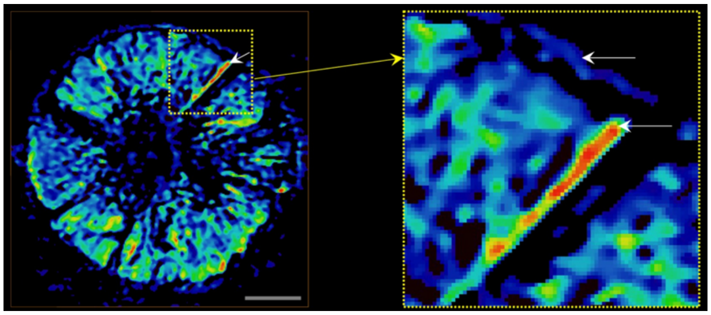

Supplementary Fig. 5. Magnification of Ni-distribution from 3D nano-tomography after cycled. Left is the figure from Figure 3c. Right is an enlarged figure of the selected area.

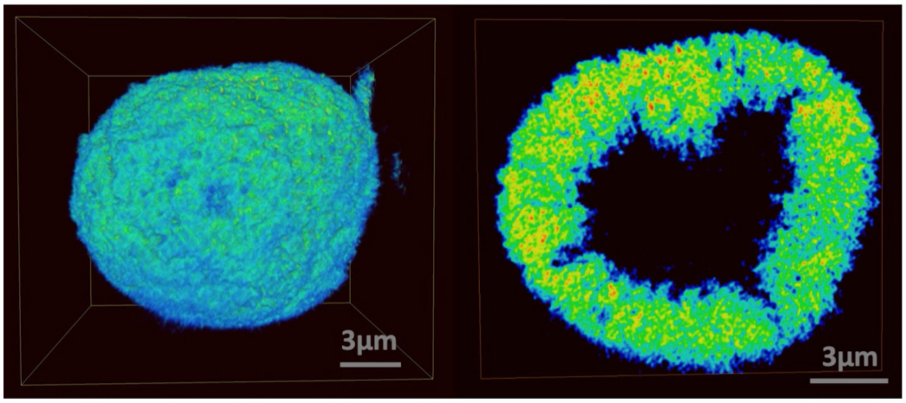

Supplementary Fig. 6. Elemental distribution observed via 3D nano-tomography. 3D Mn distribution in LR-NCM particle after cycling (left). One slide in the 3D Mn distribution (right). Scale bar: 3 µm.

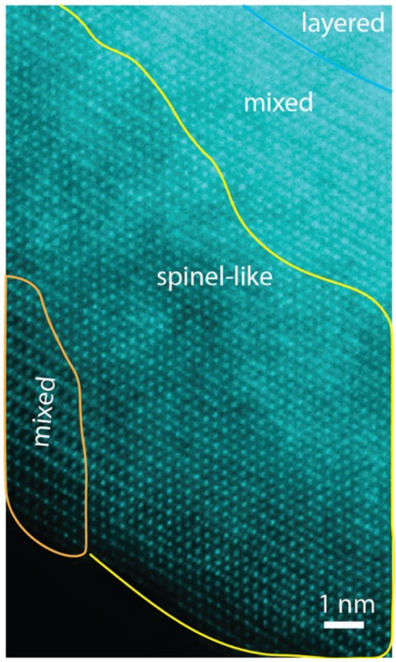

Supplementary Fig. 7. Atomic structure changes on the surface. Scale bar: 1 nm.

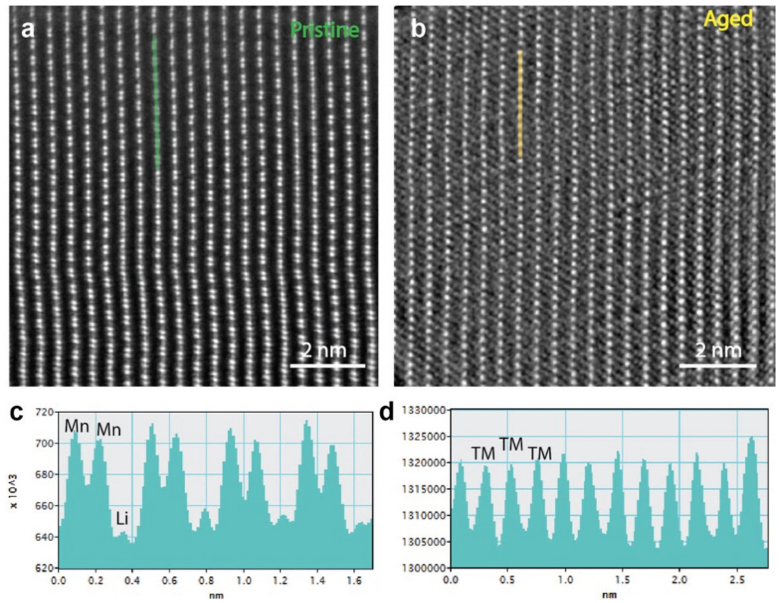

Supplementary Fig. 8. Superstructure changes over long-term cycles. STEM images of **a**, pristine and **b**, aged LR-NCM material and **c-d**, the corresponding line scan along with the marked line. Scale bar: 2 nm.

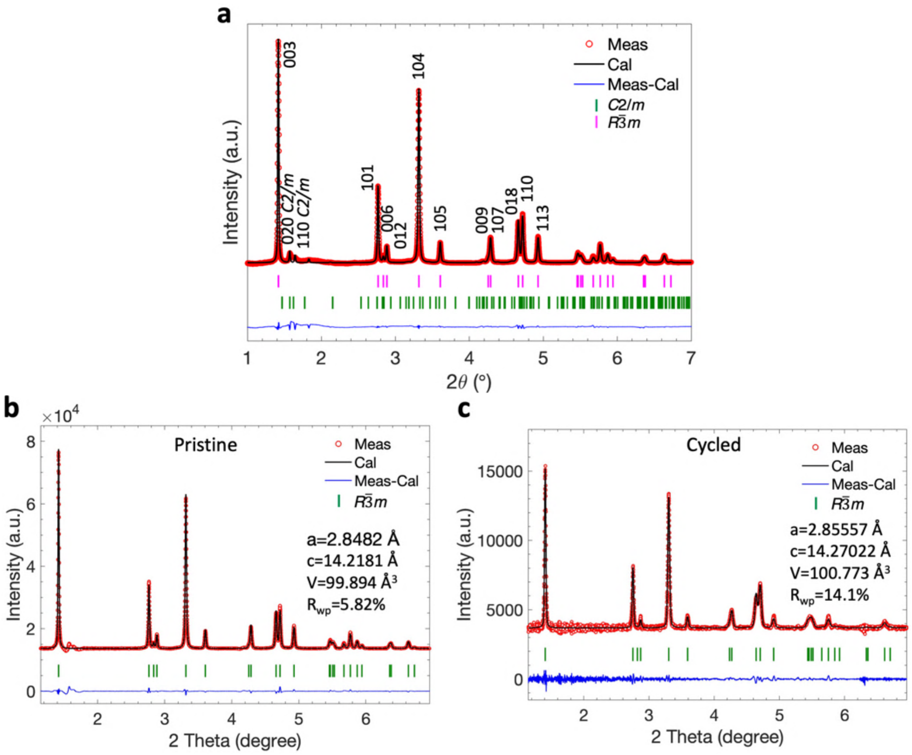

Supplementary Fig. 9. XRD Rietveld refinement of LR-NCM. a, XRD refinement results of pristine LR-NCM with a two-phase model. XRD refinement results of LR-NCM electrode **b**, at pristine state and **c**, after 200 cycles aged with one phase structural model.

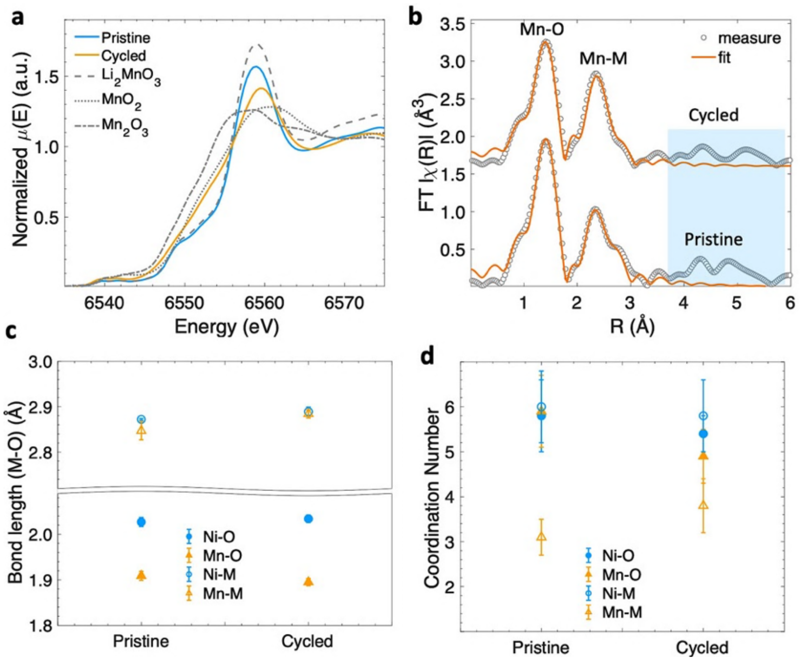

Supplementary Fig. 10. X-ray absorption spectroscopy at Mn K-edge. a, Comparison of the XANES spectra and **b,** the fitting results of Fourier transformed EXAFS curves of pristine and cycled electrodes at Mn *K*-edge. **c**, The bond lengths and **d**, coordination number of the first two shells determined by the fitting results.

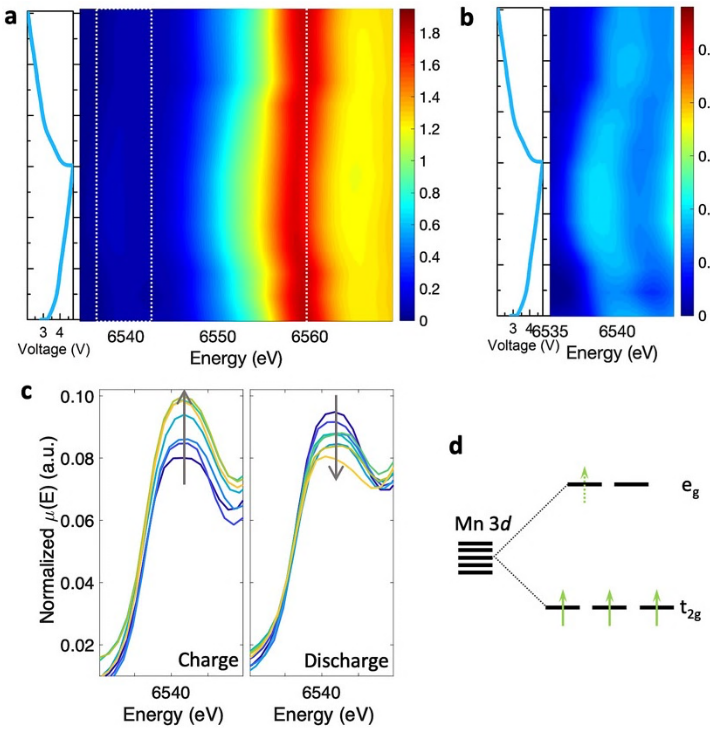

Supplementary Fig. 11. The changing reactivity of Mn. a, Contour plot of in-operando XANES spectra at Mn K-edge during the 200th cycle. **b-c,** Magnification of the pre-edge peaks. **d,** The 3*d*-electron energy level diagram for Mn^{3+}/Mn^{4+}.

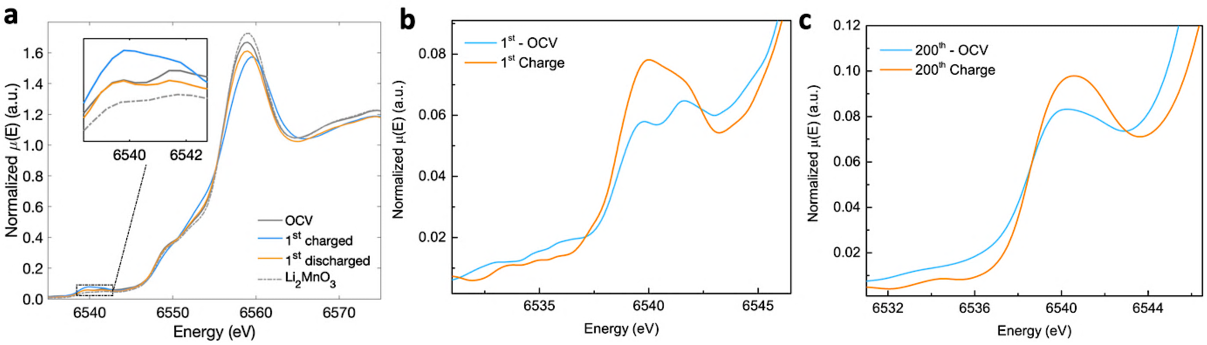

Supplementary Fig. 12. *In-situ* XANES spectra at Mn K-edge. a-b, In-situ XANES spectra during the first cycle. **c**, Pre-edge peaks evolution of Mn XANES spectra at 200th cycle.

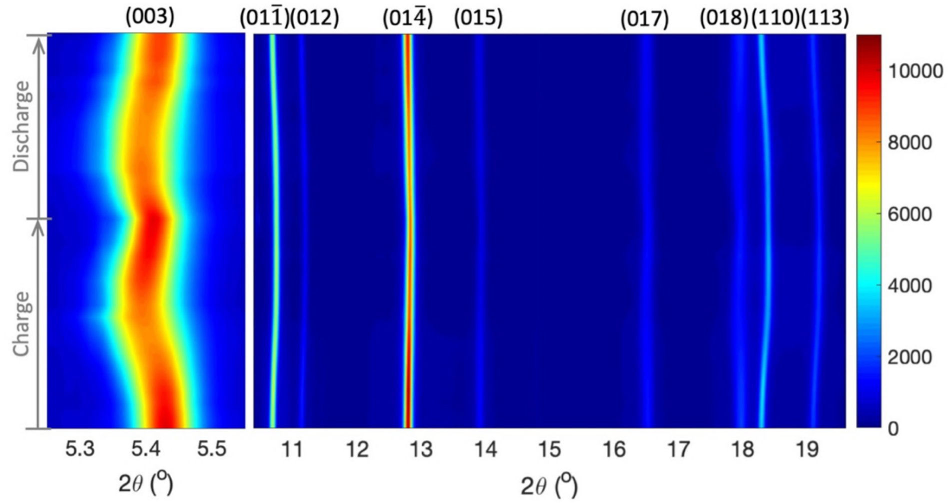

Supplementary Fig. 13. Phase transformations during the first charge-discharge process. Contour plot of *in-operando* XRD patterns during the first cycle.

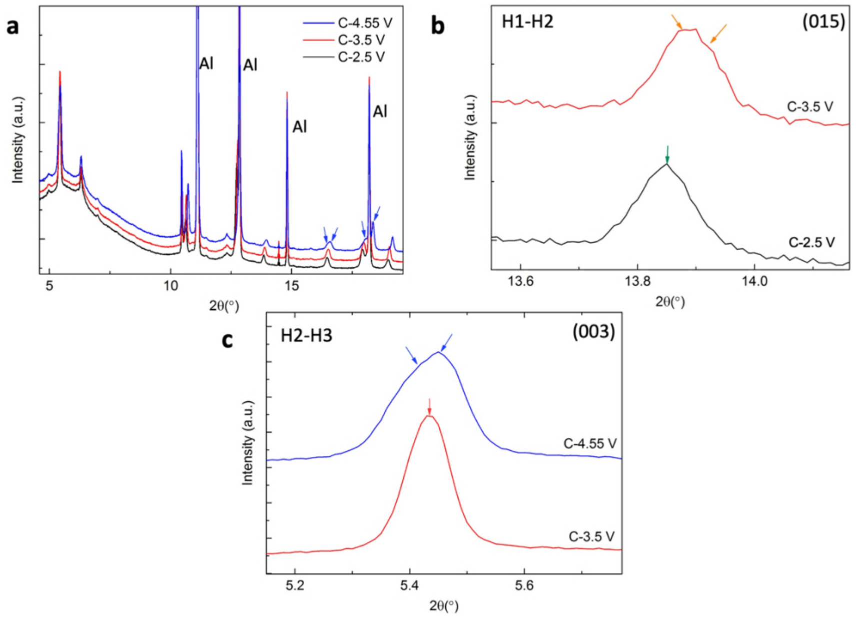

Supplementary Fig. 14. Selected *in-situ* XRD patterns at different state-of-charge during 200th charging. a. *In-situ* XRD patterns at 2.5 V, 3.5 V, and 4.55 V, respectively. Selected representative reflections of **b.** (015) and **c.** (003) to clearly show the phase transformations from H1 to H3.

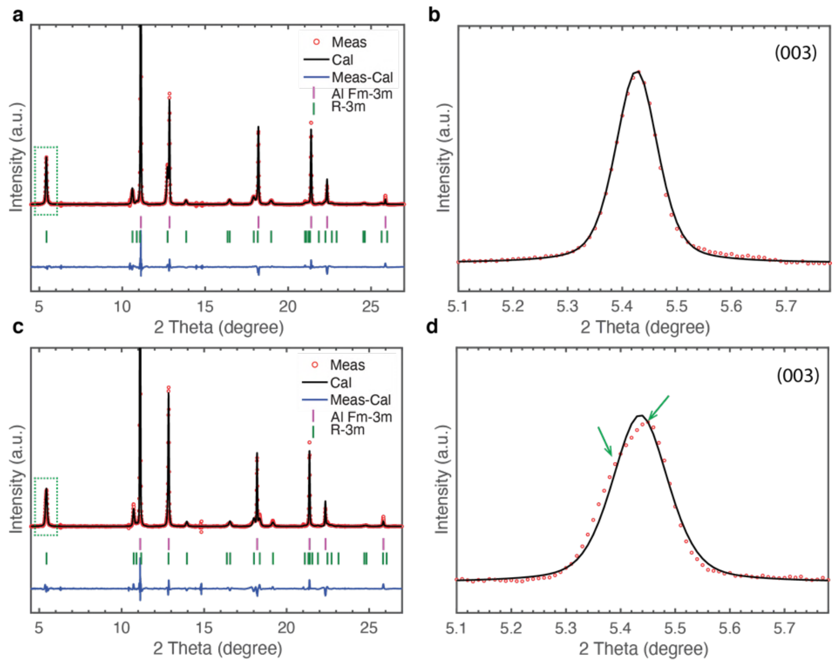

Supplementary Fig. 15. Selected Rietveld refinement results of *in-operando* XRD patterns during the 200th cycle. The XRD refinements of the LR-NCM electrode charged at **a-b**, 3.0V and **c-d**, 4.55 V.

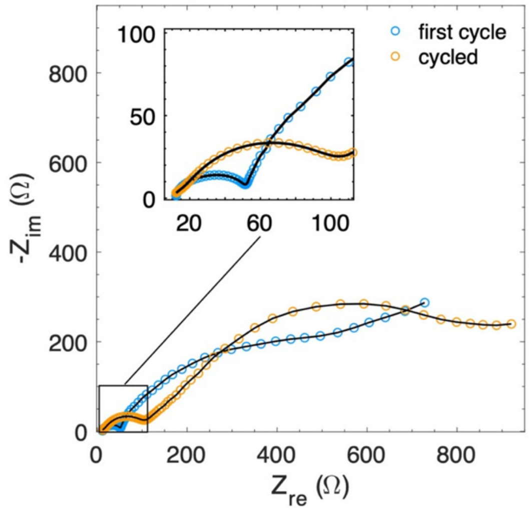

Supplementary Fig. 16. Electrochemical impedance spectroscopy (EIS) characterization. Comparison of EIS curves of LR-NCM electrode at the first cycle and after 200 cycles.

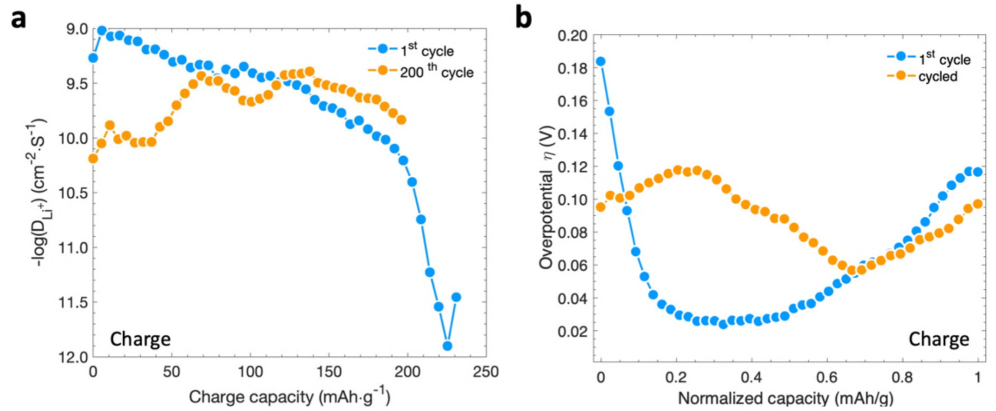

Supplementary Fig. 17. Changes of reaction kinetics. a, Corresponding D_{Li^+} and **b,** reaction overpotential (η) of the electrode at different states during the first and 200th discharge process.

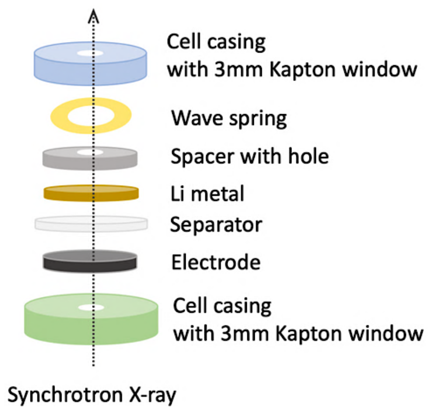

Supplementary Fig. 18. Schematic illustration of *in-operando* battery design.

Supplementary Table 1. Lattice parameters obtained by the two-phase structure model refinement of pristine LR-NCM.

**Phases**			
	**Phase fraction**	**0.57(4)**	
Phase 1		*a=b*	2.84830(1) (Å)
Space group $R\bar{3}m$	Lattice parameters	*c*	14.2183(1) (Å)
		V	99.896(1) (Å^3)
	**Phase fraction**	**0.43(4)**	
		a	4.9501(4)
Phase 2		*b*	8.5209(6)
Space group: *C2/m*	Lattice parameters	*c*	5.0250(2)
		β	109.32(4)
		V	200.05(2)
Agreement factors	Rwp (%)	8.2	
	Rp (%)	8.35	
