# Supplementary material for: Reaction inhomogeneity coupling with metal rearrangement triggers electrochemical degradation in lithium-rich layered cathode
Source: Nat Commun. 2021 Sep 10;12:5370. doi: 10.1038/s41467-021-25686-1 (PMC8433364; doi:10.1038/s41467-021-25686-1)
Supplement: Supplementary file 2 — Description of Additional Supplementary Files [file 41467_2021_25686_MOESM2_ESM.pdf]

Description of Additional Supplementary Files

Supplementary Movie 1. Inter structure for 3D nano-tomography results. The 3D nano-tomography is the same as Fig. 3a.
